# Characterization of composted sewage sludge during the maturation process: a pilot scale study

**DOI:** 10.1007/s11356-018-3335-x

**Published:** 2018-10-08

**Authors:** Marta Bożym, Grzegorz Siemiątkowski

**Affiliations:** 1 Opole University of Technology, Proszkowska 76 street, 45-758 Opole, Poland; 2 Building Materials Engineering Division in Opole, Institute of Ceramics and Building Materials, Oswiecimska 21 street, 45–641 Opole, Poland

**Keywords:** Sewage sludge, Compost, Nutrients, Heavy metals, Leachability, Stabilization degree

## Abstract

This paper determines the impact of the maturation process of composted sewage sludge on the quality of the final product and assesses the stabilization effect. The samples of composted sewage sludge were taken from a wastewater treatment plant located in Pomerania in northern Poland. The sewage sludge was composted in an open windrow composting plant with the addition of straw and wood chips in the turning windrow. The aeration of the sewage sludge mixture was conducted based on two methods. The first phase (intensive degradation phase of 6 to 8 weeks) was characterized by frequently turning; the second phase for maturation used aeration channels (2 to 3 months). In three sampling campaigns samples were taken from the same windrow after 2 (no. 1), 8 (no. 2), and 12 weeks (no. 3) of maturation. Fresh samples were used for analyzing the stabilization parameter as static respiration activity (AT_4_). Furthermore, the values of pH, organic matter (OM), total organic carbon (TOC), elementary composition, nutrients, total content, and mobile forms of heavy metals were analyzed in the compost samples. A significant decrease was found in the stabilization parameter (AT_4_) during the maturation of tested materials. In turn, no significant differences were found in the elementary composition. The concentration of most metals increased in the final product. The total content of heavy metals in the final product did not exceed the limit values for the agricultural use of sewage sludge, compost from municipal waste, and for organic fertilizers. There were no significant changes in the percentage of bioavailable and mobile forms of heavy metals during compost maturation. Zinc was characterized by the highest level of mobile and bioavailable forms, which may cause bioaccumulation after the fertilization of soil. The study has shown that the process of maturation of compost from sewage sludge not affects changes in the content of heavy metal forms. The scope of this study has been planned on a wider scale for different variants of sewage sludge composting, in order to evaluate the process.

## Introduction

According to the Polish Main Statistical Office more than 550,000 tons of dry matter (DM) of sewage sludge is produced each year in Poland (Polish Monitor 2016). In recent years, the amount of sewage sludge generated has increased following the upgrade and expansion of wastewater treatment plants in Poland. This process, which was associated with Poland’s accession to the European Union, included the fulfillment of the requirements regarding waste management. It is predicted that the total amount of sewage sludge produced in Poland will increase every year by an additional 2–3% compared to the previous year. In Poland, the preferred direction of sewage sludge utilization is associated with its natural use, including using it for agricultural purposes, land reclamation, and the cultivation of plants intended for the production of compost. The main requirement is that sewage sludge should be stabilized and sanitized before its application on agricultural land. There is an increasing tendency in Poland regarding the thermal use of sewage sludge (e.g., incineration, co–incineration pyrolysis, and gasification). In 2008, thermal use of sewage sludge was 1% compared to more than 15% in 2014. Dried sewage sludge can also be thermally utilized in cement kilns (Wzorek 2012; Yilmaz et al. 2018). The basic legal act for the agricultural use of sewage sludge in Poland is the Regulation of the Minister of the Environment dated February 6, 2015, on municipal sewage sludge (Journal of Laws 2015). This regulation describes the detailed conditions for the use of municipal sewage sludge, including dosage, range, frequency, and the use of reference analysis methods for sewage sludge and soils. The regulation specifies the maximum content of heavy metals and pathogens in the sludge but does not determine the minimum content of fertilizer components. Therefore, for the evaluation of sludge as fertilizer, the content of organic matter and macronutrients (NPK) is usually compared with the guidelines for solid organic fertilizers (Journal of Laws 2008). Apart from that, when assessing the quality of compost derived from sewage sludge in Poland, the standard concerning the quality of compost from municipal waste (BN-89/9103-090), is used.

Prior to using sewage sludge as an organic fertilizer, it should be stable and sanitized. The degree of stability is defined as the extent to which readily biodegradable organic matter has decomposed. The main biological stabilization process for the agricultural use of sewage sludge is composting. The fertilization by sludge compost improves the chemical properties of soil. Such improvements include increasing the concentration of organic matter, nutrients, and microbial biomass as well as improving physical properties, such as water holding capacity (Roig et al. 2012). These properties may be useful for the reclamation of degraded soils. However, the agricultural use of sewage sludge may be a risk to the environment due to pollution bioaccumulation and migration to groundwater (Oleszczuk 2008). Many researchers have analyzed the toxicity of heavy metals and the migration of heavy metals in soil fertilized with sewage sludge (Lopes et al. 2011; Rajmund and Bożym 2014a; Bożym and Rajmund 2015; Gattullo et al. 2017; Rajmund and Bożym 2017). In most European countries and many other countries, the heavy metals content in sludge used for agricultural purposes is limited (da Silva Oliveira et al. 2007; Gattullo et al. 2017). The total concentration of heavy metals in sewage sludge cannot provide useful information about the risk of bioavailability, toxicity, and the capacity for immobilization in the environment. It is known that the bioavailable fraction of these pollutants usually has a negative impact on the environment (Roig et al. 2012). However, the mobility and bioavailability of heavy metals in soil fertilized with compost may change over time. According to Chen (2012), during the process of composting of organic matter, humus substances can chelate heavy metals and reduce the bioavailability of these metals in the final product. However, an increase in the percentage of heavy metal mobile forms in the sewage sludge compost was observed by some authors (Oleszczuk 2008; Chen 2012; Awasthi et al. 2016). Currently, many studies are being carried out on the pollution of sewage sludge by organic compounds (Lindholm-Lehto et al. 2017). Some countries, such as Denmark, Sweden, Austria, and Germany have set standards limiting the concentration of certain organic contaminants in sewage sludge used in agriculture (Roig et al. 2012). It has been proven that the composting of sewage sludge makes it possible to reduce the contents of organic pollutants, usually polycyclic aromatic hydrocarbons (Amir et al. 2005; Lazzari et al. 2000). Moreover, composting positively influences other physical and chemical properties of sewage sludge (Oleszczuk, 2008).

Regardless of the composting method, suitable composting management is necessary to obtain a quality product (Cáceres et al. 2015). Before its agricultural use, the compost should be stabilized. Unstable compost may be problematic after application to soil due to both oxygen consumption by organic substances and the easy solubility of nutrients and pollutants. According to Yuan et al. (2016) and Sciubba et al. (2015), the application of unstable and immature compost may lead to the immobilization of nitrogen in the soil and consequently a decrease in plant growth. The stability refers to the degradation of biodegradable compounds by microbes and may be defined by the respiration activity. In the literature, many different methods have been proposed to measure compost stability based on static or dynamic methods in aerobic conditions (Iannoti et al. 1993; Gomez et al. 2006; Wagland et al. 2009; Villaseńor et al. 2011; Binner et al. 1999, 2012; Cáceres et al. 2015). Among the various analyzed tests of compost stability, the oxygen consumption rate (AT_4_, RI_4_, RA_4_, among others) has provided the most reliable values (Scoton et al. 2016). In the current study, the AT_4_ test was used to assess the stability of composts from sewage sludge during maturation. The objective of this pilot study was the assessment of the stability degree, composition, and mobility of heavy metals changes in composted sewage sludge during maturation.

## Materials and methods

### Sampling

The materials were taken from a wastewater treatment plant located in Pomerania in northern Poland. In this plant, sewage sludge is treated by composting. The material is composted in an open system with the addition of structural materials. The aeration of the sewage sludge mixture was conducted on the basis of two methods: the first phase with frequent turning and second phase for maturation using aeration channels. The quality of compost is analyzed and evaluated as fertilizer. The wastewater treatment plant has the permission of the Ministry of Agriculture to produce and sell composted sewage sludge to be applied as a fertilizer. Each year 13,000 tons of raw sewage sludge is composted; furthermore, about 7000 tons of fertilizer are produced each year.

For this investigation, effects of maturation were of interest. Thus, samples were taken only during phase 2. In three sampling campaigns, samples were taken from the same windrow after 2 (no. 1), 8 (no. 2), and 12 weeks (no. 3) of maturation according to Polish Standards (PN–EN ISO 5667–13, PN–EN ISO 5667–15, PN–Z–15011–1:1998). The composition of the feedstock mixture for the intensive phase was raw sewage sludge, straw, wood chips, and sieved recirculated compost after a previous composting process in a fresh mass ratio respectively: 16:3:4:1. The parameters of the mixture are as follows: C/N 25/1 and moisture content of about 55% WM. The height of the windrow did not exceed 1.5 m, and the volume of the windrow was over 100 m^3^. For this amount of composted material, a representative laboratory sample was collected by combining and thoroughly mixing together 30 primary samples collected at the same time from different points of the windrow. Increments were collected in rows, as is practical for windrows that are long, but not very high. The samples were collected using the probabilistic method. The samples were collected systematically (regular distance) along the entire length of the windrow in two rows: at about one third of the height and two thirds of the height. One increment was collected from about 1 m^2^ of the uncovered surface. This method of collecting samples allowed access to the entire composted material. The collected primary samples were put into a castra and mixed thoroughly. Next, the mixed material was emptied onto a prepared plot covered with foil, where the volume of the material was reduced by quartering. The laboratory samples prepared in this way were placed in tightly sealed polypropylene containers, in order to prevent loss of moisture and the influence of the environment. The samples were stored at a temperature of 4 °C during transport to the laboratory. The samples were delivered to the laboratory within 12 h. The tests for the evaluation of biological stability have to be carried out on samples of pretreated compost of sewage sludge. Thus, fresh samples were ground to obtain a < 20-mm fraction. One part of the fresh material was used for the test of biological activity and the second part of each sample was air-dried. The dried samples were ground with an agate mill and sieved and subsequently analyzed according to Polish standard PN–Z–15011–3:2001. The sampling and the TOC, CHNS, AT_4_ analyses were conducted by an accredited laboratory in Opole (ICiMB, Polish accreditation No. AB 799). The OM, pH, and heavy metal contents were analyzed in the laboratory of the Opole University of Technology.

### Biological activity test

#### Static respiration activity (AT_4_)

The AT_4_ was measured as the cumulative oxygen consumption within a particular time period, used to aerobically degrade an organic substance, reflecting the basal respiration of a material, at 20 °C and extending the duration to 4 days in mg O_2_ per g, using an OxiTop apparatus. The CO_2_ produced is measured as the CO_2_ was absorbed by NaOH and the pressure becomes negative. The AT_4_ test was carried out according to Austrian Standard (OENORM S2027–4) and a guide edited by Siemiątkowski (2012). The test was performed in triplicate.

### Physicochemical parameters

The elementary composition (CHNS) of samples was analyzed using a “Vario Macro Cube” (Elementar) with a TCD detector. Total organic carbon (TOC) was analyzed using a “Vario Macro Cube” (Elementar) with an NDIR detector. Organic matter was measured by weight loss on ignition at 550 °C in a muffle furnace for 5 h. The pH was measured in an aqueous extract (1/4, *m*/*v*) using a glass electrode with pH–conductometer CPC 501 (Elmetron) according to Polish standard PN–Z–15011–3:2001.

### Heavy metals and nutrients analysis

The compost samples were digested with the addition of aqua regia (36% HCl Tracepur® and 65% HNO3 Suprapur®, Merck) in the microwave system (Milestone, Ethos Start D). Leachable (bioavailable and mobile) forms of metals were determined by extraction tests. The bioavailable forms of metals were analyzed according to the method SM&T (Ure et al. 1993) using 0.11 M acetic acid (Emsure®, Merck). For mobile forms, an extraction with 0.5 M hydrochloric acid (0.5 M HCl) (Tracepur®, Merck) was used.

The analytical procedure 40 mL of a 0.11 M Ac solution was added to 1 g ± 0.001 g of a dry material, and the mixture was shaken for 16 h at room temperature. This method is applied to measure the leaching of metals in the ion–exchangeable and carbonate forms. Acetic acid is a non-specific acid and may be applied for leaching metals bound to silicates and carbonates. Moreover, mobile forms of the metals were analyzed using a 0.5 M hydrochloric acid (0.5 M HCl) (Tracepur®, Merck) according to the Polish Chemical and Agricultural Stations procedure was used based on the Polish Standard (PN–R–04024:1997). The analytical procedure 100 mL of a 0.5 M HCl solution was added to 2 g ± 0.001 g of dry material; the mixture was shaken for 30 min, then left for 24 h without shaking and filtered. Both extractions were carried out at room temperature (20–25 °C). All metals (Cd, Pb, Cu, Zn, Cr, Ni, Mo, Co, Fe, Mn, Mg, Ca, K, Na) content measurements were made with a Solaar 6 M (Thermo) using the FAAS technique.

### Quality control

For quality control, *the certificate materials* were used, respectively, *CHNS* and *TOC analysis* Alfalfa B2273 Cert. No. 41505 and heavy metals “Trace metals—Sewage amended soil” CRM005–50G (Sigma–Aldrich, Lot: LRAB1009). The uncertainty of measurements was presented as a relative expanded uncertainty *U* (%) for coverage factor *k* = 2, confidence level *α* = 95%. The relative expanded uncertainty (*U*) was calculated for sampling and for each analytical method, based on the uncertainty budget. The uncertainty budget was calculated on the basis of inter-laboratory tests (proficiency tests), the results of CRM, and spiked samples analysis. The *U* value for each method is as follows: sampling including sample preparation 5%; OM 12%; pH 5%; C 7%; H 18%; N 16%; S 16%; TOC 15%; AT_4_ 15%; Cd, Pb, Cu, Zn, Cr, Ni, Mo, Co, and Mn 10%; Fe 12%; Ca 10%; Mg 13%; K 8%; and Na 10%. The current results of the analyses were presented as an average value calculated with a minimum of triplicate assays and a relatively expanded uncertainty (*U*) calculated for each parameter.

## Results

The results of the physicochemical analyses, with AT_4_ and the total content of heavy metals and nutrients in the tested materials are presented in Table 1. The percentages of mobile forms eluted with 0.5 M HCl (PN–R–04024:1997) and bioavailable forms eluted with 0.11 M Ac (Ure et al. 1993) are presented in Table 2. Table 3 shows the limit values of heavy metals concentration for sewage sludge amended soil, solid organic fertilizers, and first class (the highest quality) of compost from municipal waste according to Polish standards.

**Table 1:** Characteristic of samples: after 2 weeks (no. 1), 8 weeks (no. 2), and 12 weeks (no. 3) of maturation process; the mean value (± *U*) of each parameter

Parameter	No. of sample			*U* [%]
	No. 1	No. 2	No. 3	
OM [% DM]	60 (± 7)	57 (± 7)	55 (± 7)	12
pH_H2O_	7.5 (± 0.4)	7.3 (± 0.4)	7.0 (± 0.4)	5
C [% DM]	37 (± 3)	38 (±3)	30 (± 2)	7
H [% DM]	4.8 (± 0.9)	4.9 (± 0.9)	3.9 (± 0.7)	8
N [% DM]	3.1 (± 0.5)	3.0 (± 0.5)	3.9 (± 0.6)	16
S [% DM]	0.6 (± 0.1)	2.5 (± 0.4)	0.9 (± 0.1)	16
TOC [% DM]	33 (± 5)	30 (± 5)	28 (± 4)	15
AT_4_ [mgO_2_ g^−1^ DM]	19.8 (± 3.0)	6.5 (± 1.0)	4.6 (± 0.8)	15
Cd [mg kg^−1^ DM]	0.89 (± 0.09)	0.81 (± 0.08)	0.79 (± 0.08)	10
Pb [mg kg^−1^ DM]	28 (± 3)	33 (± 3)	36 (± 4)	10
Cu [mg kg^−1^ DM]	61 (± 6)	65 (± 7)	69 (± 7)	10
Zn [mg kg^−1^ DM]	585 (± 59)	581 (± 58)	676 (±68)	10
Cr [mg kg^−1^ DM]	24 (± 2)	28 (± 3)	31 (± 3)	10
Ni [mg kg^−1^ DM]	12 (± 1)	12 (± 1)	14 (± 1)	10
Mo [mg kg^−1^ DM]	< 1	< 1	< 1	10
Co [mg kg^−1^ DM]	5.7 (± 0.6)	6.7 (± 0.7)	8.3 (± 0.8)	10
Fe [mg kg^−1^ DM]	883 (± 106)	1640 (± 197)	4254 (± 510)	12
Mn [mg kg^−1^ DM]	51 (± 5)	97 (± 10)	228 (± 23)	10
Ca [% DM]	6.2 (±0.6)	5.6 (± 0.6)	6.4 (± 0.6)	10
Mg [% DM]	1.5 (± 0.2)	1.3 (± 0.2)	1.3 (± 0.2)	13
K [% DM]	0.4 (± 0.0)	0.4 (± 0.0)	0.3 (± 0.0)	8
Na [% DM]	0.1 (± 0.0)	0.1 (± 0.0)	0.1 (± 0.0)	10

*OM* organic matter, *C* carbon, *H* hydrogen, *N* nitrogen, *S* sulfur, *TOC* total organic carbon, *AT4* static respiration activity index, *U* relative expanded uncertainty for *k* = 2 and *α* = 95%

**Table 2:** The concentration of mobile (0.5 M HCl) and bioavailable (0.11 Ac) forms of metals (mean ± *U*) and percentage of eluted forms calculated taking into account total content

Metal	No. of sample						*U* [%]
	No. 1		No. 2		No. 3		
	0.5 M HCl	0.11 M Ac	0.5 M HCl	0.11 M Ac	0.5 M HCl	0.11 M Ac	
Cd [mg kg^−1^ DM]; [% of total conc.]	0.13 (± 0.01); 15	< 0.1; –	0.16 (± 0.02); 20	< 0.1; –	0.18 (± 0.02); 23	< 0.1; –	10
Pb [mg kg^−1^ DM]; [% of total conc.]	5.9 (± 0.6); 21	1.2 (± 0.1); 4	< 1.0; –	2.0 (± 0.2); 6	< 1.0; –	1.6 (± 0.2); 4	10
Cu [mg kg^−1^ DM]; [% of total conc.]	14 (± 1); 23	2.0 (± 0.2); 3	11 (± 1); 17	1.4 (± 0.1); 2	5.9 (± 0.6); 9	1.2 (± 0.1); 2	10
Zn [mg kg^−1^ DM]; [% of total conc.]	339 (± 34); 57	142 (± 14); 24	354 (± 35); 60	143 (± 14); 25	350 (± 35); 52	115(± 12); 17	10
Cr [mg kg^−1^ DM]; [% of total conc.]	3 (± 0); 12	< 1; –	4 (± 0); 13	< 1; –	4 (± 0); 13	< 1; –	10
Ni [mg kg^−1^ DM]; [% of total conc.]	3 (± 0); 24	2 (± 0); 14	3 (± 0); 27	2 (± 0); 12	3 (± 0); 24	1 (± 0); 9	10
Mo [mg kg^−1^ DM]; [% of total conc.]	< 1; –	< 1; –	< 1; –	< 1; –	< 1; –	< 1; –	10
Co [mg kg^−1^ DM]; [% of total conc.]	1.8 (± 0.2); 32	< 1; –	2.1 (± 0.2); 31	1.0 (± 0.1); 15	2.1 (± 0.2); 25	< 1; –	10
Fe [mg kg^−1^ DM]; [% of total conc.]	417 (± 50); 47	77 (± 9); 9	488 (± 59); 29	54 (± 6); 3	489 (± 59); 11	74 (± 9); 2	12
Mn [mg kg^−1^ DM]; [% of total conc.]	14 (± 1); 27	7 (± 1); 15	28 (± 3); 29	13 (± 1); 13	42 (± 4); 18	22 (± 2); 10	10

*DM* dry matter, *Ac* acetic acid, *U* relative expanded uncertainty for coverage factor *k* = 2, confidence level *α* = 95%

**Table 3:** Heavy metals concentration limits according to Polish standards for sewage sludge amended soil, solid organic fertilizers, and first class means the highest quality of compost from municipal waste

Metal	Limit of heavy metals for			The value of teste compost after 12 weeks of maturation****
	Sewage sludge used in agriculture and for land reclamation in agricultural purposes*	Solid organic fertilizer**	The first class of compost from municipal waste quality***	
Cd [mg kg^−1^ DM]	20	5	5	0.79 (± 0.08)
Pb [mg kg^−1^ DM]	750	140	350	36 (± 4)
Cu [mg kg^−1^ DM]	1000	No limitation^a^	300	69 (± 7)
Zn [mg kg^−1^ DM]	2500		1500	676 (± 68)
Ni [mg kg^−1^ DM]	300	60	100	14 (± 1)
Cr [mg kg^−1^ DM]	500	100	300	31 (± 3)
Hg [mg kg^−1^ DM]	16	2	No limitation^b^	No data

*According to Journal of Laws 2015

**According to Journal of Laws 2008

***According to Polish Standard BN–89/9103–090

****The directions of using tested compost based on the total content of heavy metal: (1) in agriculture, as the cultivation of all agricultural products placed on the market, including crops for the production of feed; (2) for growing plants intended for the production of compost; (3) for the cultivation of plants not intended for consumption and for the production of feed; (4) for the reclamation of land, including land for agricultural purposes, for adaption of land to specific needs resulting from waste management plans, spatial development plans

^a^Cu and Zn content in organic fertilizers is not limited (Journal of Laws 2008)

^b^Hg content in compost from municipal waste is not limited (BN–89/9103–090)

## Discussion

The initial pH value of 2-week-old (no. 1) maturated materials was 7.5 (± 0.4) and decreased to 7.0 (± 0.4) over the 3 months (no. 3) of maturation. A decrease in the pH of the tested materials was probably caused by nitrate formation that implies H^+^ release during nitrification (Cáceres et al. 2018). Biological nitrification causes the oxidation of ammonia to nitrates, which consequently reduces the pH of the compost (Castaldi et al. 2008). This is one of the explanations for this phenomenon, because the nitrate content has not been analyzed in the current study. Furthermore, the pH parameter is very relevant to minimize nitrogen losses (Bernal et al. 2009). Usually pH is not the main factor for the assessment of the composting process. However, according to Oviedo-Ocaña et al. (2015), the pH of the composted biowaste was considered as a maturity parameter. The authors suggest that to assess the stability and maturity of the compost, the following parameters are also used: the respirometric index, germination index (GI), C/N ratio, self-heating, volatile solids and on-site methods, such as temperature, odor, color, etc. (Oviedo-Ocaña et al. 2015).

The percentage of OM decreased during the maturing process from 60 (± 7) % DM (no. 1) to 55 (± 7) % DM (no. 3). The concentration of OM in sewage sludge was closely related to TOC content. During the maturation process, TOC content decreased over time from 33 (± 5) to 28 (± 4) % DM. In a study of sewage sludge after treatment, Smidt and Parravicini (2009) also reported a reduction in the percentage of TOC from 39% DM in primary sludge to 26.7% DM in anaerobically digested sludge and finally to 23.2% DM after aeration. Likewise, Pognani et al. (2011) observed changes in the TOC content in composted sewage sludge from 50.7% DM for the input to 39% DM for the output composting tunnels after 3 weeks of processing. In the current study, a reduction in the total carbon content of the samples was also observed. No significant changes in H, N, and S content during the maturation process of the tested materials was found; the content of these elements varied over time (Table 1).

### Stability degree

Aerobic respiration indices are usually highlighted as the most suitable tools to monitor the composting process, mainly for stability and maturity assessment (Mejias et al. 2017). The stability level of compost is one of most important aspects representing the quality of the composting process (Lasaridi and Stentiford 1998). The Austrian Landfill Ordinance regulates the limit values for the degree of stabilization of waste (allowance for landfilling, not for compost) to a Respiration Activity (AT_4_) < 7 mg O_2_ g^−1^ DM (Binner et al. 2012). There are no regulations in Poland for assessing the degree of stabilization of landfilled waste.

Moreover, there are no such tests in Poland to assess the stability of compost used for natural purposes. Therefore, in the present study, the Austrian standards for landfilled waste after MBT were used. For comparison reasons, these parameters were determined for the three stages of the degree of sludge stabilization. It is evident that composted sewage sludge does not reach the limit values for landfilling in Austria, but those parameters could be useful to determine the degree of its stabilization. According to Roig et al. (2012), for sewage sludge, the value of a 4-day oxygen uptake is lower than the 30 mg O_2_ g^−1^ DM; the authors considered it as a stable organic matter, while results above 80 mg O_2_ g^−1^ DM they considered to be raw organic matter. The authors received the results of respiration activity tests, which had the lowest values for compost additionally processed under anaerobic conditions and thermally dried sewage sludge; the values were close to the stable organic matter category. The authors suggest that microbial degradation of the most labile organic fractions is higher during the composting process of sewage sludge than during the other treatments.

In the current study after 2 weeks of maturation (no. 1), the sample was characterized by high respiration activity AT_4_ with 19.8 (± 3.0) mg O_2_ g^−1^ DM. During maturation, the value of the AT_4_ parameter decreased, respectively: after 2 months, AT_4_ was 6.5 (± 1.0) mg O_2_ g^−1^ DM and after 3 months, it was 4.6 (± 0.8) mg O_2_ g^−1^ DM. Based on the results of the AT_4_ parameter analysis, we may conclude that the 3-month maturation of sewage sludge has reduced the value of the AT_4_ parameter to a level that results in the sufficient stabilization of the materials. For comparison, Sidełko et al. (2017) found a decrease in the AT_4_ value from 26.29 mg O_2_ g^−1^ DM at the first day of composting to 3.05 mg O_2_ g^−1^ DM after 95 days of sewage sludge aerobic treated in compost windrow. The authors investigated a composted sewage sludge with straw and wood chips. Composting was carried out in a windrow during 6 weeks. The first stage of composting was carried out with intensive aeration. This stage lasted 6 weeks, then the material matured in windrow up to the 3 months. The authors marked a significant decrease in AT_4_ values already on day 17 of the first phase of composting (value about 10 mg O_2_ g^−1^ DM). However, they did not explain this effect. In comparison, Pognani et al. (2011) determined the value of AT_4_ for the composted material (sewage sludge and wood chips) in composting tunnels before the process to be 111 mg O_2_ g^−1^ DM, and after 3 weeks of treatment, it was 15 mg O_2_ g^−1^ DM. Mejias et al. (2017) measured AT_4_ in fresh, non-digested sewage sludge at 312 mg O_2_ g^−1^ DM, while for cow manure, it was (241 mg O_2_ g^−1^ DM) and pig slurry (209 mg O_2_ g^−1^ DM). The authors were expecting lower values of AT_4_ for cow manure and pig slurry as compared to sludge, because although these are normally biologically active wastes, they can lose respiration activity during storage on farms. Smidt and Parravicini (2009) presented the results of respiration activity (RA_4_) analysis for sewage sludge after treatment: the primary sludge was at a level of 285 mg O_2_ g^−1^ DM, excess sludge from the aerobic activated sludge tank after thickening 113 mg O_2_ g^−1^ DM, sludge after anaerobic treatment 77 mg O_2_ g^−1^ DM, and the same sludge with additional aeration was at 70 mg O_2_ g^−1^ DM. Additionally, the authors also analyzed the gas forming potential (gas sum = GS_21)_ in treated sewage sludge. They found that RA_4_ and GS_21_ are correlated in aerobically treated samples. However, they no found a correlation between those parameters for sewage sludge treated under anaerobic conditions.

### Heavy metals and nutrients

Some of the metals are classified as trace elements, essential for the development of soil microorganisms and plants (Kabata-Pendias and Pendias 1999). For most heavy metals (Cd, Pb, Hg), no biological properties have been identified. Adequate metal concentrations stimulate soil microbial growth. However, high levels of these heavy metals can reduce the effectiveness of or completely inhibit the biological activity of microorganisms in aerobic/anaerobic conditions (Kabata-Pendias and Pendias 1999; Bożym et al. 2015). On the other hand, the nutrient ions (Na, K, Mg, Ca) regulate the pH of soil and they are essential for microbial growth and, similar to other nutrients, they affect the specific growth rates of bacteria in soil and plants. Regular agricultural application of sewage sludge or products made from waste may lead to a progressive accumulation of heavy metals in soil and crops (Chen et al. 2010; Lopes et al. 2011; Sciubba et al. 2015; Rorat et al. 2016; Gattullo et al. 2017). In the current study, the results of the metals content survey for the samples are given in Table 1.

The current study demonstrated a relatively low *cadmium* concentration in the tested materials, as the measured values varied between 0.79 (± 0.08)–0.89 (± 0.09) mg kg^−1^ DM. Cd concentration in the analyzed samples was higher than the values found by Villaseńor et al. (2011) in a composted mixture of sewage sludge with straw or sawdust: 0.11–0.12 mg kg^−1^ DM. In contrast, Gattullo et al. (2017) reported a cadmium content of 0.5 mg kg^−1^ DM in Italian sewage sludge compost. A wide range of Cd was observed by Oleszczuk (2008) in a study of compost from Polish sewage sludge 2–76 mg kg^−1^ DM. The *lead* content of the tested materials ranged from 28 (± 3) to 36 (± 4) mg kg^−1^ DM. Gattullo et al. (2017) obtained similar results of lead content in composted sewage sludge (29 mg kg^−1^ DM), while Oleszczuk (2008) obtained a higher content range of 35–52 mg kg^−1^ DM. Villaseńor et al. (2011) found a lower lead content in compost mixtures (48.6 and 53.2 mg kg^−1^ DM) compared to the final product of sewage sludge composting (113 mg kg^−1^ DM). The *zinc* and *copper* contents of the tested materials were: Zn 581 (± 58)–676 (± 68) mg kg^−1^ DM and Cu 61 (± 6)–69 (± 7) mg kg^−1^ DM, respectively. A study by Oleszczuk (2008) recorded higher contents of both metals in Polish composted sewage sludge.

It was in the range of Zn 935–1490 and Cu 155–314 mg kg^−1^ DM. In contrast, in a study of Italian sewage sludge compost, Gattullo et al. (2017) analyzed concentrations of Zn 302 mg kg^−1^ DM and Cu 128 mg kg^−1^ DM. Villaseńor et al. (2011) indicated a higher content of both metals in the final product of composted sewage sludge and straw/sawdust (Zn 877 and Cu 336 mg kg^−1^ DM) compared to substrate mixtures before treatment: Zn 433–438 and Cu 214–242 mg kg^−1^ DM. *Nickel* content in the tested materials ranged between 12 (± 1) and 14 (± 1) mg kg^−1^ DM. This range is lower in comparison to the result recorded by Oleszczuk (2008) in composts from Polish sewage sludge: 18–178 mg kg^−1^ DM. A higher nickel content in a composted mixtures with sewage sludge and waste was determined by Villaseńor et al. (2011) 43.4–49.7 mg kg^−1^ DM; and in the final product 87.8 mg kg^−1^ DM. In the studies of Gattullo et al. 2017, the nickel content in a compost from sewage sludge was 37 mg kg^−1^ DM. In the tested materials, chromium content ranged from 24 (± 2) to 31 (± 3) mg kg^−1^ DM. The Cr concentrations in the analyzed samples was slightly lower than the values found by Oleszczuk (2008) in Polish composts from sewage sludge, i.e., from 26 to 125 mg kg^−1^ DM. Furthermore, the study by Villaseńor et al. (2011) registered the results of chromium content in sewage sludge compost mixtures as ranging from 34 to 38.6 mg kg^−1^ DM; in the final product, it was 66.3 mg kg^−1^ DM. The latter was similar to the figure found in Italian composted sewage sludge: 64 mg kg^−1^ DM (Gattullo et al. 2017). Limit values for the content of other metals, such as *Mo*, *Co*, *Mn*, and *Fe* in Polish sewage sludge used for agricultural purposes, composts from municipal wastes, and organic fertilizers have not been determined. However, da Silva Oliveira et al. (2007) suggested that safety levels should be established for other metals in cases of sewage sludge used in agriculture. In Polish sewage sludges, the content of Co is usually contained within the 2–40 mg kg^−1^ DM limit; and rarely exceeds a content of 25 mg kg^−1^ DM (Bożym and Rajmund 2015). In the tested material, cobalt ranged between 5.7 (± 0.6) and 8.3 (± 0.8) mg kg^−1^ DM and the molybdenum content was below the limit of determination (< 1 mg kg^−1^ DM). According to Rajmund and Bożym (2014b), the content of molybdenum in sewage sludge (SS) and their composts (SSC) was respectively 2.80 and 2.18 mg kg^−1^ DM. The authors also analyzed the cobalt content in these samples, and they obtained results in the range 6.7 mg kg^−1^ DM (SS) and 6.0 mg kg^−1^ DM (SSC) (Bożym and Rajmund 2015). Gattullo et al. (2017) analyzed Co at a level of 5.4 mg kg^−1^ DM in Italian sewage sludge compost. Because *iron* and *manganese* are characterized by low toxicity, Polish regulations do not standardize the content of these metals in sewage sludge used in agriculture and in fertilizers. Rajmund and Bożym (2017) determined the content of Fe and Mn in sewage sludge (SS) and compost (SSC) from a small Polish treatment plant to be in the range of Fe 3348 mg kg^−1^ DM (SS) and 3241 mg kg^−1^ DM (SSC) and Mn 106 mg kg^−1^ DM (SS) and 138 mg kg^−1^ DM (SSC), respectively. In sewage sludge from a wastewater treatment plant in Madrid, Walter et al. (2006) found a large range of iron content (9330–16,390 mg kg^−1^ DM) depending on the treatment method, as compared to a low range of manganese: 143–215 mg kg^−1^ DM. In the current study, the tested materials comprised a wide range of iron and manganese concentrations: Fe 883 (± 106)–4254 (± 510) and Mn 51 (± 5)–228 (± 23) mg kg^−1^ DM. A very strong increase in the concentrations of both of these metals in the final product after the maturation process was observed. It is difficult to assess this phenomenon, because the increase was larger than can be explained by degradation of organic matter. The composted materials were probably contaminated with those metals during mechanical processing, such as turning, screening, and aerating.

*Nutrients* such as Na, K, Mg, and Ca are essential for plant and microbial growth in soil. Potassium, along with nitrogen, and phosphorus are considered to be macronutrients. Sewage sludge is usually characterized by a low concentration of those elements. In addition, Ca and Mg are often referred to as secondary nutrients. They are also necessary for the growth of plants, but they mainly regulate the pH and affect the availability of nutrients in the soil (Gorlach and Mazur 2002). The optimum content of sodium in the soil affects the adequate course of processes performed by soil microorganisms and stimulates the development of plants. The high concentration of sodium affects soil salinity and levels of water absorption by plants. In the current study, a nutrients contents of the tested materials were as follows: Ca 5.6 (± 0.6)–6.4 (± 0.6) % DM; Mg 1.3 (± 0.2)–1.5 (± 0.2) % DM; K 0.3 (± 0.0)–0.4 (± 0.0) % DM; and Na 0.1 (± 0.0) % DM. By comparison, Roig et al. (2012) found that Ca was the most abundant nutrient (3.9–9.5%), and the lowest percentage nutrient in the tested sewage was potassium (0.2–0.6%). The authors noted no significant differences between sludge treatments and nutrients content. They found that the content of nutrients depends not only on the sewage sludge treatment efficiency but also on the sources of the sewage.

In summary, the results of this work are in accordance with the ranges for composted sewage sludge reported by other authors. The total content of metals in sewage sludge depends primarily on the source of wastewater (municipal, industrial) and their composition and less on the treatment of sewage sludge. For comparison, Roig et al. (2012) presented a wide range of heavy metal concentrations in sewage sludge from the output of 24 urban wastewater treatment plants in Spain. The authors classified sludge samples into five different main types according to the sludge treatment and post-treatment processes. They found significant differences in metal concentrations within the sludge samples from the same and between the different categories, due to the different kinds of effluents discharged into sewers, i.e., Cd 1.1–13; Pb 27–123; Cu 131–1456; Zn 2.5–2292; Cr 38–518; and Ni 16–410 mg kg^−1^ DM. In current study, heavy metal content increased in composted sewage sludge during maturation. Most of these heavy metals in sewage sludge are retained in the compost after processing. The increase in heavy metals is due to the mineralization of organic matter, this leads to a higher concentration at the end of the process (Lazzari et al. 2000; Cai et al. 2007; Liu et al. 2007). However, in this project, there were no significant changes in the content of nutrients in the compost during maturation. This may suggest that metals, such as Ca, Mg, K, and Na may have been leached, probably due to the high water solubility of their compounds.

### Forms of metals

The application of sewage sludge or compost may lead to increases in the amounts of mobile forms of heavy metals in soil. Treatment with sewage sludge has been shown to affect heavy metal mobility. Metal availability may either increase or decrease due to liming, the fermentation process, or composting/vermicomposting, among others, depending on the type of treatment of sewage sludge (Abdel-Shafy and Mansour 2014; Milinovic et al. 2014; Rajmund and Bożym 2014a; Bożym 2016; Bożym and Bok 2016; Rorat et al. 2016). The speciation of heavy metals in sewage sludge during the composting process may depend on its initial chemical composition, also on its treatment and on the organic matter transformations during composting. Although the composting process should reduce the leachability of heavy metals, due to absorption by humus (Paré et al. 1999; Cai et al. 2007), in some cases, an increase in the percentage of mobile forms in compost or vermicompost from sewage sludge has been observed (Amir et al. 2005; Walter et al. 2006; Liu et al. 2007; Rajmund and Bożym 2014a; Bożym 2016; Bożym and Bok 2016; Ingelmo et al. 2012). This effect could be caused a suboptimal rotting process where mineralization dominated against humification (enhanced decomposition of organic matter and missing re-binding of mobile forms to the structure of humus).

The extraction performed by the application of 0.11 M acetic acid (Ac) affects the leaching of the ion-exchangeable metal fraction and carbonate fractions, as these forms can be released from sewage sludge to soil and are bioavailable. In the current study, a wide range of metal concentrations leached with 0.11 M Ac was found. The highest level of bioavailability was observed with regard to zinc (17–25% of total amount) (Table 2). A similar percentage value was observed for nickel and manganese: Ni 9–14% and Mn 10–15%, respectively. The percentage of bioavailable forms of Pb, Cu, and Fe was low (2–9%). The lowest content of Cd and Co in bioavailable forms were analyzed, in some cases below the limit of quantification. The 0.11 M Ac extraction released bioavailable forms of metals in the following order: Zn > Mn,Ni > Fe > Pb > Cu.

A slight decrease in the percentage of mobile (0.5 M HCl) forms of metals, except cadmium, in samples during the maturation process was observed. Hydrochloric acid is an aggressive eluent and causes the leaching of available forms, associated with carbonate and organic matter forms of metals. It may leach both bioavailable and also some bounded metal forms. The metal leaching sequence with 0.5 M HCl in the tested samples was as follows: Zn (52–60%), Co (25–32%), Cd (15–23%), Mn (18–29%), Fe (11–47%), Cu (9–23%), Pb (0–21%), and Cr (12–13%) (Table 2). In the current research, it was found that zinc was characterized by the highest mobility (52–60%). A similar mobility of zinc was obtained in previous studies of composts and vermicomposts from sewage sludge (40–75%) (Bożym 2016; Rajmund and Bożym 2014a). A high percentage of zinc may suggest its high mobility in soil fertilized with the tested compost.

A different order of leaching for 0.5 M HCl extraction as compared to 0.11 M Ac was obtained, respectively: Zn > Fe > Co,Mn,Ni > Cu,Pb > Cd > Cr (no. 1) and Zn > Ni,Co,Cd > Cr,Mn > Fe,Cu > Pb (no. 3). According to the study of Rajmund and Bożym (2014a), the leaching of metals with hydrochloric acid from raw sludge (SS) and its compost (SSC) from a rural wastewater treatment plant was in the following order: Zn > Cd > Cu > Pb > Ni > Cr.

### Polish regulation

The limit values for the concentration of heavy metals in sewage sludge used for agriculture, solid organic fertilizers, and composts from municipal wastes imposed by Polish regulations are reported in Table 3. According to one of the regulations, the compost analyzed within this project could be classified as class I–III depending on its quality including heavy metal contents (BN–89/9103–090). This classification allows to assess the quality of compost produced from municipal waste, but it is also used by sewage treatment plants to assess the quality of composts from sewage sludge. Classification according to the BN standard indicates the degree of contamination of composts, but does not described the directions of those use. Many producers of composts from sewage sludge refer to the guidelines of this standard in quality certificates. This standard allows very high heavy metal contents in the first class of composts. Significantly lower metal contents are allowed in composts used for fertilizer purposes in other countries. When compared to Poland, the maximum allowed concentrations of heavy metals in compost are lower in Holland, Germany, or Spain. The values for each metal in these countries are, respectively, Cd 0.7, 1.5, and 0.7; Pb 65, 150, and 45; Cu 25, 100, and 70; and Zn 75, 400, and 200 mg kg^−1^ DM (Chen et al. 2010; Villaseńor et al. 2011). However, the Polish standard (BN–89/9103–090) is not the main legal act allowing compost to be traded. The main legal act allowing for the sale of compost from organic waste, including sewage sludge, as a “fertilizer” is the Act on fertilizers and fertilization (Journal of Laws 2008). In accordance with the Act, the entities launching fertilizers produced on the basis of organic substances need to acquire appropriate permission from the Polish Minister of Agriculture.

The Act defines the scope of research and requirements, which make it possible to give a permit for launching such a fertilizer. In this Act, heavy metal content limits in fertilizers are low (Table 3). However, for composts from sewage sludge used for purposes other than as a “fertilizer,” e.g., as strengtheners in plant cultivation or for reclamation, quality standards set out in other legal acts are used, including a trade standard (BN–89/9103–090) or a sewage sludge regulation (Journal of Laws 2015) (Table 3). In comparison, according to an Italian directive on fertilizers, the maximum admissible limits for organic amendments are respectively Cd 1.5; Cu 260; Ni 100; Pb 140; Zn 500; and Cr (VI) 0.5 mg kg^−1^ DM (Gattullo et al. 2017). In contrast, Brazil and the USA have higher allowable limits of heavy metals in sewage sludge used for agriculture: Cd 85; Cu 4300; Ni 420; Pb 840; and Zn 7500 mg kg^−1^ DM (da Silva Oliveira et al. 2007). The content of heavy metals in the tested samples was lower than the limit values for sewage sludge used in agriculture, solid organic fertilizer, and the first class of quality of compost from municipal waste according to Polish law.

## Conclusions

The results of this study show that the maturation process of composting sewage sludge has an impact on the properties of the final product, such as organic matter and total organic carbon concentrations, total heavy metals, and nutrients. On the other hand, there was no significant effect of the maturation of the compost on changes in the percentage of mobile and bioavailable forms of the metals. Unfortunately, an analysis of the raw sewage sludge was not carried out, which would allow an evaluation of the differences within the final product. The low total content of heavy metals in the tested materials ensures their use in agriculture as fertilizer. It has been shown that composted sewage sludge after maturation is stable, which may indicate the sufficient stabilization of the organic matter of the tested materials. The AT_4_ parameter is an important indicator that may be used to assess the degree of stabilization in a properly conducted composting process. However, only three samples were used in the current study, which is an insufficient number to draw far-reaching conclusions. These pilot studies will be continued on a wider scale for different variants of sewage sludge composting, in order to evaluate the process.
